# Assessment of brown adipose tissue function

**DOI:** 10.3389/fphys.2013.00128

**Published:** 2013-06-04

**Authors:** Sam Virtue, Antonio Vidal-Puig

**Affiliations:** ^1^Metabolic Research Laboratories, Addenbrooke's Treatment Centre, Institute of Metabolic Science, Addenbrooke's Hospital, University of CambridgeCambridge, UK; ^2^Wellcome Trust Sanger InstituteCambridge, UK

**Keywords:** brown adipose tissue, BAT, maximal thermogenic capacity, adipose tissue, energy expenditure, cold exposure

## Abstract

In this review we discuss practical considerations for the assessment of brown adipose tissue in rodent models, focusing on mice. The central aim of the review is to provide a critical appraisal of the utility of specialized techniques for assessing brown adipose tissue function *in vivo*. We cover several of the most common specialized methods for analysing brown adipose tissue function *in vivo*, including assessment of maximal thermogenic capacity by indirect calorimetry and the measurement of sympathetic tone to brown adipose tissue. While these techniques are powerful, they are not readily available to all laboratories; therefore we also cover several simple measurements that, particularly in combination, can be used to determine if a mouse model is likely to have alterations in brown adipose tissue function. Such techniques include: pair feeding, analysis of brown adipose tissue lipid content and mRNA and protein markers of brown adipose tissue activation.

## Introduction

The aim of this review is to discuss the practical considerations of current methods for assessing brown adipose tissue (BAT) function in rodents, focusing largely on mice. While it is impossible to ignore the theoretical concepts behind assessment of BAT, these were recently covered in a comprehensive review (Cannon and Nedergaard, 2011).

BAT is a thermogenic organ and it acts to generate heat in order to maintain thermal homeostasis. As will be discussed below, nutrient oxidation in BAT can account for over 60% of the total energy expenditure of the mouse. Given the enormous effect of BAT on metabolic rate, alterations in BAT activity can impact on multiple different metabolic variables and therefore understanding the effect of BAT is essential for any branch of murine metabolic phenotyping.

## The impact of cold exposure on different metabolic parameters

Mice housed at 5°C exhibit metabolic rates two and half times higher than mice acclimated to 30°C. This dramatic increase in metabolic rate can impact on multiple aspects of metabolism. Cold exposure can render mice resistant to diet induced obesity (Cannon and Nedergaard, 2009). Additionally, the rate of insulin-independent glucose disposal in cold acclimated mice is so high it can prevent the manifestation of hyperglycaemia in streptozotocin treated rats, a type 1 diabetic model (Takano et al., 1987). Remarkably, there is at least one case report of systemic administration of thyroid hormone, which leads to BAT activation, being able to overcome the effects of a partial loss of function mutation in the insulin receptor (type A insulin resistance) in humans (Skarulis et al., 2010). In addition to its impacts on carbohydrate metabolism, cold exposure can: (1) affect nervous control of the cardiovascular system (Swoap et al., 2008), (2) confound the interpretation of activity data from calorimetric studies (Virtue et al., 2012a), and (3) normalize serum triglyceride levels in Apoa5-null mice (Bartelt et al., 2011). Given that 60% of the calories consumed by wild-type, cold-acclimated mice are oxidized in BAT, the potential for BAT to mediate cold-induced changes in carbohydrate and lipid metabolism is substantial and must be considered when interpreting data regarding whole-organism metabolic changes.

## How does BAT generate heat?

In order to understand how cold exposure and BAT activity can have such a dramatic impact on all aspects of metabolism it is necessary to understand the function of BAT. BAT is a thermogenic organ and its principal function is to convert nutrients to heat. The production of heat by BAT is also called non-shivering thermogenesis (NST). In order to generate heat, BAT possesses a unique protein called uncoupling protein 1 (UCP1). UCP1 acts to uncouple oxidative phosphorylation from ATP production. While its exact molecular mechanism is still a topic of debate, UCP1 allows protons to pass from the mitochondrial intermembrane space into the mitochondrial matrix. Activated UCP1 therefore sets up a futile cycle where the electron transport chain (ETC) pumps protons across the inner mitochondrial membrane and UCP1 allows them to flow back into the mitochondrial matrix. Crucially, UCP1 activity dissipates the inner mitochondrial membrane potential, which normally acts to limit the ETC. Therefore, the theoretical potential for BAT to oxidize nutrients is limited only by its nutrient supply and capacity for oxidative metabolism and, in line with this fact, BAT has the highest metabolic rate of any organ.

The term “BAT” is in some regards a relatively arbitrary description. Almost all adipose tissue depots in the mouse contain a mixture of brown and white adipocytes (Murano et al., 2009). Importantly, the relative balance of brown and white adipocytes can be dramatically increased or decreased by environmental and pharmacological interventions.

In recent years, it has become apparent that brown adipocytes located in different depots are derived from different populations of precursors. Brown adipocytes located in canonical BAT depots are suggested to come from a precursor that is common to brown adipocytes and muscle (Timmons et al., 2007; Seale et al., 2008). Conversely, brown adipocytes located in predominantly white adipose tissue (WAT) depots are suggested to share a precursor with white adipocytes. Brown adipocytes found in WAT depots have distinct transcriptional profiles from canonical brown adipocytes and have therefore been classed as a separate cell type, known as brite or beige cells, to reflect their intermediate profile between canonical brown and white adipocytes.

## Impact of temperature on metabolic rate

The specific generation of heat may at first glance seem a relatively simple function for an organ, particularly given that heat is a by-product of most metabolic processes. However, virtually all aspects of BAT have evolved to maximize its capacity to oxidize nutrients. In an animal housed at 5°C, 60% of all the energy expended by a mouse is done so in BAT (Golozoubova et al., 2004; Cannon and Nedergaard, 2011). To allow such high oxidative rates BAT requires a substantial blood supply in order to provide nutrients and oxygen and to carry away waste products and heat. To that end, cold exposure causes extensive angiogenesis within BAT (Xue et al., 2009). In addition to specific changes in BAT in response to cold exposure, metabolic changes necessary for the mouse to adapt to cold exposure occur in multiple other organs. Animals increase their heart weight and increase their cardiac output to provide more blood flow in order to supply nutrients to either BAT (for NST) or muscle for shivering (Shechtman et al., 1990).

In contrast to cold exposure, heat production by BAT falls to almost nothing at thermoneutrality. The thermoneutral zone is defined as a temperature at which an organism does not have to employ active heat production nor evaporative heat dissipation to maintain its core body temperature. In mice the thermoneutral zone falls between 28°C and 33°C dependent on strain, gender and age. Below the thermoneutral zone an organism must expend energy to generate heat. This may be generated through processes such as shivering in muscle or NST, believed to occur principally in BAT. Above the thermoneutral zone animals must use energy to actively reduce their body temperature. Processes such as sweating, panting or saliva spreading on fur can promote heat loss to a lesser or greater extent, dependent on the organism.

Below the thermoneutral zone, in a C57Bl/6 mouse, energy expenditure increases by 8% per 1°C drop in environmental temperature (relative to energy expenditure at thermoneutrality) (Virtue et al., 2012a). Thus, a mouse expending 0.33 W at 30°C will expend 0.66 W at 18°C and 1 W at 5°C. The increase in energy expenditure in response to a reduction in environmental temperature appears to be linear, at least as far as 5°C. This has important implications when considering “room temperature” experiments in mice. Standard laboratory conditions range from 20 to 24°C, therefore a 30 g mouse housed at 24°C could be expected to expend approximately 0.5 W, whereas at 20°C it would be expected to expend 0.6 W. It is worth noting that 0.1 W is a 20% difference in metabolic rate—a difference larger than the impact of most genetic manipulations. Therefore, it is critical when considering any metabolic study to both control the environmental temperature and to state it as accurately as possible to allow reproduction of results.

## The sympathetic nervous system

Importantly, BAT activity is able to rapidly adapt to changes in environmental temperature. A sudden shift in environmental temperature from 5 to 30°C is met by a very rapid and proportionate reduction in energy expenditure in order to prevent hyperthermia. Unsurprisingly, these rapid adaptations are under nervous control. The principal arm of the nervous system that regulates BAT activity is the sympathetic nervous system (SNS).

The SNS is the single most important regulator of BAT function (Cannon and Nedergaard, 2004). The SNS regulates both acute BAT function and also prolonged BAT adaptation. The principal adrenergic receptor responsible for activating mature brown adipocytes is the β3-adrenergic receptor. The β3-adrenergic receptor also promotes differentiation of brown adipocytes that have been cultured for 6 days (Rehnmark et al., 1990; Bronnikov et al., 1999). On brown preadipocytes that have been cultured for up to 3 days the β1-adrenergic receptor appears to be the most prominent receptor and mediates noradrenergic stimulation of brown adipocyte proliferation (Bronnikov et al., 1992, 1999; Cannon and Nedergaard, 2004).

Activation of the β3-adrenergic receptor on mature brown adipocytes causes a rise in intracellular cyclic adenosine monophosphate (cAMP). This in turn activates lipolysis via protein kinase A (PKA)-mediated phosphorylation of the lipid droplet-associated protein perilipin (Souza et al., 2007). Phosphorylation of perilipin releases the protein Comparative Gene Identification-58 (CGI-58) which in turn activates adipose triglyceride lipase (ATGL) (Granneman et al., 2007, 2009). ATGL predominantly catalyses the break down of triglyceride to diglyceride (Zimmermann et al., 2004). Elevated lipolysis increases intracellular free fatty acid (FFA) levels, which activate UCP1. The absence of ATGL renders mice profoundly cold-intolerant, either due to insufficient FFAs for UCP1 activation, or due to a failure to mobilize FFAs from lipid droplets within BAT and WAT to provide substrates for fatty acid oxidation (Haemmerle et al., 2006). In addition to ATGL, hormone sensitive lipase (HSL) is also activated by PKA-mediated phosphorylation (Miyoshi et al., 2007). HSL appears to be less critical for thermal homeostasis than ATGL, with mice lacking HSL exhibiting normal cold tolerance (Osuga et al., 2000). However, HSL is directly phosphorylated in response to β-adrenergic stimulation by PKA on a number of serine residues (Holm, 2003), making HSL phosphorylation a useful molecular marker of BAT activation. When sympathetic tone to BAT falls, lipolysis diminishes and FFA levels fall, reducing UCP1 activation. Purinergic nucleotides can subsequently bind to UCP1 and inactivate it (Nicholls, 1974), allowing a rapid switching off of BAT thermogenesis.

In addition to the stimulatory role of the β3-adrenergic receptor, the α2-adrenergic receptor acts to inhibit the response of BAT in the presence of norepinephrine. The α2-adrenergic receptor is G_i_ coupled, and its stimulation lowers intracellular cAMP levels. Inhibition of α2-adrenergic receptors actually increases cAMP levels and the expression of UCP1 in brown adipocytes when they are stimulated with norepinephrine (Bronnikov et al., 1999).

## Acute vs. adapted cold exposure

BAT undergoes extensive remodeling in response to cold, with this remodeling taking 3–5 weeks in mice. Cold exposure leads to a pronounced increase in BAT mass via adipogenesis and an increase in mitochondrial density. Total UCP1 protein mass increases in BAT during acclimation to the cold and overall thermogenic capacity is increased (Nedergaard and Cannon, 2013). Cold acclimation also promotes BAT angiogenesis (Xue et al., 2009) and also increases adipose tissue sympathetic nerve fiber density (Murano et al., 2009). Therefore, when studying mice with alterations in BAT function, it is important to consider at which stage of thermogenic adaptation an animal was when interpreting results from metabolic experiments. Warm or cold **exposure** is usually used to indicate an acute change in temperature lasting up to perhaps 72 h. Importantly, during cold exposure, BAT will be in the process of dynamic remodeling to either increase or decrease its thermogenic capacity. Conversely, cold or warm **acclimation** usually refers to a period of time of between 7 days and 3 months of housing at a constant temperature. After acclimation, BAT is assumed to have reached a steady state with respect to its level of thermogenic capacity. It is important to note that BAT will not be fully cold acclimated until between 3 and 5 weeks after moving from a room temperature (20–24°C) to a cold environment (5°C), therefore in some “cold acclimation” studies, BAT may still be undergoing remodeling.

A further complexity comes from the fact that reports investigating the impact of short-term exposure to either cold or warm environments often use different prior acclimation temperatures, which will affect the thermogenic capacity of mice. Additionally, studies also expose mice to different final temperatures, which will also affect the degree of BAT activation.

In general the majority of published literature regarding both exposure and acclimation experiments focuses on four major temperature ranges; thermoneutrality (28–33°C) when BAT activity is assumed to be negligible; “standard laboratory housing conditions” (20–24°C); cold exposure (usually 4–5°C), probably chosen as it is the temperature most commercial refrigerators maintain; and 18°C, in part used as an intermediate temperature chosen because (1) some mouse strains will not tolerate the transfer from 30 to 5°C, and (2) because it allows the detection of intermediate phenotypes that cannot be distinguished by shifts from 30 to 24°C or 30°C to 5°C (Golozoubova et al., 2001).

Overall, when considering any study, an appreciation of the environmental temperature an experimental animal was housed at prior to the experiment commencing and, if different, the temperature the study was conducted at is essential for interpretation of the results.

## Shivering vs. non-shivering thermogenesis (NST)

Full cold acclimation takes between 3 and 5 weeks dependent on mouse strain and magnitude of the thermal challenge (Nedergaard and Cannon, 2013). When a mouse is transferred from environments above 18°C to the cold (5°C), the thermogenic capacity of BAT is initially insufficient to maintain core body temperature, so heat must be generated from other sources. The principal acute source of thermogenesis in the mouse is shivering. Electromyography (EMG) traces show strong elevations in nervous tone to muscle when mice are first exposed to cold (Golozoubova et al., 2001). Over the first 4 weeks of cold exposure, EMG readings fall as BAT takes over heat generation from muscle. After 1 month of cold exposure, cold acclimated mice have similar EMG readings to mice that are housed at thermoneutrality, indicating that BAT is fully capable of maintaining thermal homeostasis. Notably, the UCP1 KO mouse, which cannot generate heat in BAT, maintains an elevated EMG reading even after 1 month of cold acclimation, supporting the concept that these mice principally use shivering thermogenesis to generate heat, even in a chronic setting (Golozoubova et al., 2001).

## Common factors that induce BAT activity

### Diet

The concept of luxuskonsumption, now called diet-induced thermogenesis (DIT), was first identified in the nineteenth century based on the observation that over eating in both humans and dogs was associated with lower rates of weight gain than expected given the increase in caloric intake. In the 1970s Rothwell and Stock discovered that DIT in rats was associated with a series of metabolic changes in BAT, consistent with increased thermogenesis (Rothwell and Stock, 1979, 1983). Feldman et al. have demonstrated that UCP1 KO mice housed at thermoneutrality have greater weight gain on a high-fat diet than wild-type mice (Feldmann et al., 2009). This result provides the first conclusive evidence that BAT is a site of DIT. An important unresolved question is how high-fat feeding leads to the activation of adaptive thermogenesis.

### Temperature

As already mentioned, temperature is the single biggest factor affecting BAT function and overall metabolic rate. For mice housed below thermoneutrality, the increase in energy expenditure per degree centigrade decrease in environmental temperature has been estimated to be between 6% (Herrington, 1940) and 8% (Virtue et al., 2012a).

### Drugs

A large number of drugs have been shown to modify energy expenditure and alter BAT function. The compounds that most directly affect BAT activity are those targeting the β-adrenergic system. Direct activation of β3-adrenergic receptors by agents such as CL316243 increases energy expenditure. In addition to their effects on energy expenditure β3-adrenergic agonists also promote brown adipocyte differentiation (Cannon and Nedergaard, 2004). Additionally, the thiazolidinedione class of drugs target the nuclear hormone receptor peroxisome proliferator-activated receptor gamma (PPARγ) and promote the browning of WAT in rodents (Sell et al., 2004). A second critical nuclear hormone receptor for BAT development is the thyroid hormone receptor (Silva, 2006). Mice lacking the enzyme deiodinase 2 (Dio2), which is necessary for the conversion of T4 to the active T3 form in BAT, have impaired cold tolerance. Furthermore, lack of Dio2 was associated with reduced lipolysis and oxygen consumption in isolated brown adipocytes (de Jesus et al., 2001). Thyroid hormone replacement therapy in humans has also been associated with browning of WAT (Skarulis et al., 2010). Drugs that modulate the SNS, including cocaine, amphetamines (Kong et al., 2003) and ephedrine (Baba et al., 2007), also have known thermogenic actions. Serotonergic drugs can increase BAT activity by promoting the release of catecholamines from adrenergic neurons in BAT (Steiner and Evans, 1976). Nicotine has also been demonstrated to exert some of its weight reducing effects through activation of BAT (Martínez de Morentin et al., 2012). Overall, when using any drug which has weight-lowering actions, a potential effect on BAT should be considered and/or assessed.

### Insulation

Insulation is perhaps one of the single most important variables when considering energy balance. A lack of comprehension regarding the impacts of skin barrier dysfunction or hair-loss can lead to the misinterpretation of results. Perhaps the best example of how skin barrier function can affect metabolic results is the Stearoyl-CoA desaturase 1 (SCD1) KO mouse. Much of the phenotyping of the SCD1 KO mouse focused on the fact that SCD1 KO mice were resistant to diet induced obesity and had improved insulin sensitivity. Importantly, the SCD1 KO mouse has a dramatic loss of fur, leading to very poor thermal insulation. Consequently at any given temperature below thermoneutrality, the SCD1 KO mouse is under a much greater cold stress than a wild-type mouse (Ntambi et al., 2002). In order to meet the greater thermal challenge, SCD1 mice have to generate more heat than wild-type controls and as a result are hyper-metabolic (Binczek et al., 2007). As mentioned above, elevated metabolic rate is able to protect against many metabolic disorders, including diet-induced obesity. Of note, mice which only lack SCD1 in liver and thus have normal skin barrier function were not resistant to high-fat diet-induced obesity, but still exhibited improved insulin sensitivity (Miyazaki et al., 2007).

## Calorimetry—a powerful tool for measuring BAT function

The principal product of BAT is heat. As such, calorimetry represents probably the single most useful tool for assessment of BAT function *in vivo*. Indirect calorimetry by gas exchange is the most common form of calorimetry used to measure energy expenditure in rodents. Gas-exchange indirect calorimetry relies on calculating energy expenditure from the consumption of oxygen (O_2_) and production of carbon dioxide (CO_2_) by an organism.

### Calculating energy expenditure from oxygen consumption and CO_2_ production

Energy expenditure (EE) can be calculated from the amount of O_2_ consumed (called VO_2_) and the amount of CO_2_ produced (VCO_2_) using the Weir equation (Equation 1). The amount of energy produced per mole of oxygen consumed varies dependent on substrate by approximately 6%, with fatty acid oxidation producing less energy per mole of oxygen than carbohydrate oxidation. Therefore, when assessing metabolic rate it is necessary to calculate energy expenditure rather than just relying on VO_2_, particularly when there are changes in substrate utilization (for example when switching from a high carbohydrate diet to a high-fat diet, or when analysing mice that are undergoing fasting, where endogenous lipid stores are oxidized).

(1) $$\text{EE}(J)=15.818{\text{VO}}_{2}+5.176{\text{VCO}}_{2}$$

A critical aspect of energy expenditure calculations is that VO_2_ and VCO_2_ are not equal under most conditions. The oxidation of different macronutrients results in the production of different quantities of CO_2_. The ratio VCO2/VO2 is known as the respiratory exchange ratio (RER) or respiratory quotient (RQ) and can provide information about substrate oxidation. The RERs for the three major macronutrients are 1 for carbohydrate, 0.7 for lipid and approximately 0.9 for protein, dependent on amino acid composition. If considering RER only in terms of nutrient oxidation, then RER would be expected to range from 1 (pure carbohydrate oxidation) to 0.7 (pure lipid oxidation).

### Interconversion of nutrients and respiratory exchange ratio

While RER predominantly provides information about nutrient oxidation, several other metabolic processes can impact on the RER of an organism, leading to RER values which exceed the ranges possible purely from the oxidation of nutrients. In practice RER values are often observed which exceed 1, particularly when mice are fed a diet high in carbohydrate (such as laboratory chow diets which usually have a carbohydrate content of 60% or more). The process of *de novo* lipogenesis, by which carbohydrate is converted to lipid, has an RER of approximately 5. While *de novo lipogenesis* will only ever account for a small amount of all CO_2_ production, it is not uncommon to observe RER values of 1.1 during periods when mice are synthesizing lipid.

A second point regarding RER is that it can be affected by changes in energy balance. Animals which are losing fat mass will have lower RERs than if they are weight-stable. When in negative energy balance, mice will rapidly utilize all their glycogen and begin oxidizing fat, which will reduce their RER relative to a weight-stable animals as oxidizing fat has an RER of 0.7. When considering differences between two groups of animals (for example a group of wild-type mice and a group of knock-out mice) it is important to consider any changes in body weight that occur between the groups while they are being measured. If one group of animals has lost more weight than the other they will almost certainly exhibit a reduction in RER, however, this reduction may be secondary to a change in energy balance, as opposed to a primary effect of altered lipid or carbohydrate handling.

### Impact of BAT on the metabolic rate of free-living animals

As mentioned previously, metabolic rate is greatly increased by reductions in ambient temperature. Following full cold acclimation, heat production in wild-type mice largely occurs in BAT, therefore any differences in cold induced energy expenditure compared to controls is indicative of altered BAT function. However, it is important to realize that an increase in metabolic rate *per se* does not necessarily indicate an increase in BAT activity. UCP1 KO mice lack the ability to generate heat in BAT by uncoupled respiration, and predominantly use muscle shivering to maintain core body temperature (Golozoubova et al., 2001, 2006). Loss of BAT function, however, does not reduce total daily energy expenditure, with UCP1 KO mice having been demonstrated to have a metabolic rate at least as high as wild-type mice in multiple studies (Inokuma et al., 2006; Ukropec et al., 2006; Meyer et al., 2010). Therefore, an assessment of metabolic rate in response to changes in ambient temperature only indicates an alteration in thermogenesis but is not specific for BAT function.

## Assessment of maximal thermogenic capacity

A better measure than total daily energy expenditure for assessing BAT function is to consider maximal thermogenic capacity. Maximal thermogenic capacity refers to the greatest quantity of heat that a mouse *can* produce, as opposed to how much heat it produces under free-living conditions. To assess maximal thermogenic capacity a supramaximal dose of a thermogenic drug (usually norepinephrine or the β3-adrenergic receptor agonist CL316243) is administered to an animal to maximally activate BAT. Inevitably, organs other than BAT will also be stimulated in response to adrenergic agonists. In order to identify BAT-specific alterations in thermogenic capacity it is necessary to measure maximal thermogenic capacity at two separate temperatures. First, maximal thermogenic capacity is measured after mice have been acclimated to thermoneutrality to minimize BAT thermogenic capacity. Second, mice are acclimated to 4°C in order to produce a large increase in BAT thermogenic capacity. Ideally the same group of animals will be acclimating sequentially to the two different temperatures, with maximal thermogenic capacity assessed after each acclimation. Alternatively, two separate groups of animals can be used. Cannon and Nedergaard have demonstrated that the difference between norepinephrine stimulated energy expenditure at 4°C and at 30°C is entirely dependent on the presence of UCP1 (Golozoubova et al., 2006), suggesting that this component of thermogenic capacity is BAT dependent. As such, assessment of maximal thermogenic capacity provides a relatively direct measure of BAT function that can be conducted on living animals. Importantly, the actual measurement of thermogenic capacity must be conducted at thermoneutrality in order to turn off the sympathetic nervous tone, regardless of prior acclimation.

**Figure 1:**
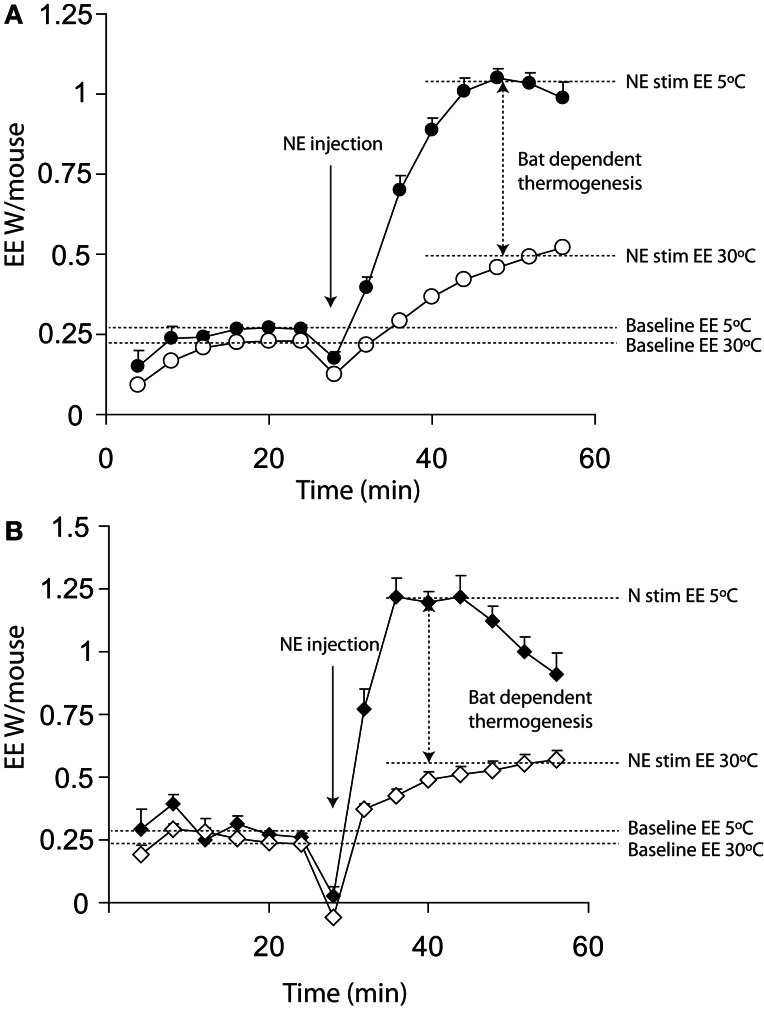
**Energy expenditure from 11 warm acclimated (30°C for 3 weeks) and 8 cold acclimated (5°C for 3 weeks) mice.** Mice were injected with 1 mg/kg norepinephrine at the time point indicted. All mice were anaesthetized with 60 mg/kg pentobarbital and measured at 30°C, regardless of prior acclimation temperature. **(A)** Energy expenditure uncorrected for lag showing a successive increase in energy expenditure between 0 and 16 (prior to injection but following the mouse being place in the chamber) minutes and 24 and 40 min (post injection with norepinephrine). **(B)** The same data as shown in panel **(A)** corrected for lag, showing apparently more rapid changes in energy expenditure. All mice; male, C57Bl/6, 4 months of age, chamber volume 2.7 l. Energy expenditure measurements conducted using a custom built calorimetry system, using a paramagnetic oxygen analyser and infrared carbon dioxide detector. Flow rate was 0.4 l/min measured on incurrent air to the chambers.

Figure 1A shows a typical plot of maximal thermogenic capacity for wild-type mice that have been acclimated to either 5°C or 30°C for 3 weeks prior to measurement. The energy expenditure dependent on BAT is indicated by the vertical arrow between the two norepinephrine stimulated (right hand side) portions of the plots.

This profile of change in energy expenditure is additionally influenced by two critical factors; the impact of lag and whether the animals are conscious or unconscious (anaesthetized). Failure to consider these factors can significantly affect the results.

## The impact of lag

**Figure 2:**
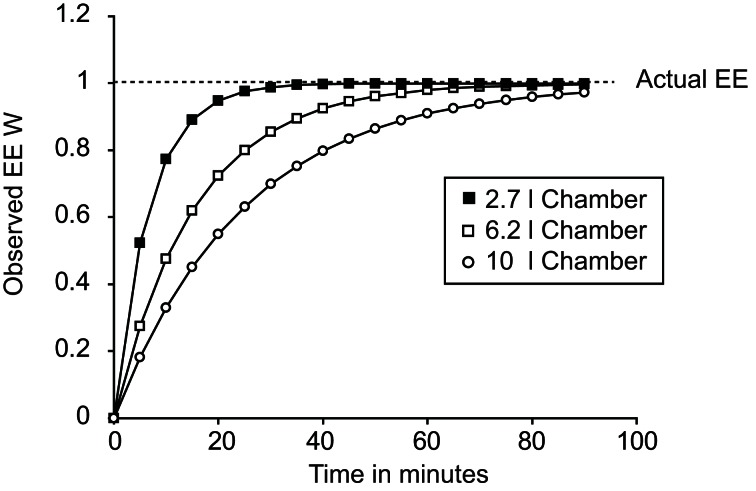
**Plot demonstrating the effect of calorimetric lag for different chambers.** All cases show the theoretical effect of lag on observed changes in energy expenditure dependent on chamber volume. In all three cases a “mouse” was placed into the chamber at time 0 expending 1 W. Chamber volumes of 2.7 l, 6.2 l, and 10 l correspond to the volumes of a Columbus mouse Oxymax chamber, Columbus CLAMS mouse double-feeder cage and the approximate volume of a Tecniplast mouse cage now used in home-cage calorimetry systems.

A key factor in any assessment of maximal thermogenic capacity is lag. A lag is a non-specific delay between two events occurring. In the case of calorimetry, lag refers to the time between energy being expended by a mouse and the change in energy expenditure being observed. Importantly, the lag is not a simple linear delay that can be corrected for by realigning data. Figure 2 demonstrates the energy expenditure that would be observed if a theoretical mouse producing 1 W of energy was placed into chambers of different sizes with an air flow of 0.4 l/min. The larger the chamber, the longer it takes between a change in energy expenditure occurring and the system coming to equilibrium.

In theory a calorimetry chamber should show a monoexponential relationship between changes in the rate of consumption or production of gases by a mouse and the change in the observed gas concentration within the chamber. The likelihood is that a monoexponential model is an over simplification as calorimeters have multiple separate chambers potentially including the calorimetry chamber itself, tubing between chambers and analysers, drying chambers, and measurement chambers. However, in most systems the calorimetry chamber volume predominates and most of the lag can be accounted for by a simple monoexponential correction.

## Correcting for lag

Correcting for lag employs an approach called the instantaneous or Z correction (Lighton, 2008). The idea behind the correction is to consider not only the observed energy expenditure at any given time point, but also the rate of change in energy expenditure between the current observation and the previous observation. Mathematically the approach combines the gas concentration observed at a given time point (T_0_) and adds to it the derivative of the gas concentrations between the current time point and the previous time point (T_0_–T_1_), multiplied by a constant. The constant is related to the chamber volume, the flow rate and the time between observations, so a small change in a larger chamber will equate to a large change in smaller chamber. In theory this approach allows energy expenditure values to be generated that represent the actual energy expenditure at any given time point. The following section provides a brief description of how to correct for lag, however, a much more detailed description of the calculations for both energy expenditure and lag correction are provided by Lighton (2008).

Most commercial calorimetry systems do not automatically provide lag corrected energy expenditure. Before explaining how to correct for lag, it is necessary to explain how to derive VO_2_ and VCO_2_, as these equations are necessary in order to apply lag correction. To calculate the amount of oxygen consumed by the mouse (the VO_2_) then the fractional concentration (Fc) of the oxygen entering and leaving the chamber must be known, as must the flow rate (FR) entering and leaving the chamber. Fractional concentrations range between 0 and 1, whereas gas concentrations in most commercial calorimetry systems are usually described as percentages. Percentages can be converted to fractional concentrations by dividing by 100. The equation for VO_2_ is shown below in Equation 2:

(2) $${\text{VO}}_{2}={\text{FR}}_{\text{in}}{\text{FcO}}_{2\text{in}}-{\text{FR}}_{\text{out}}{\text{FcO}}_{2\text{out}}$$

In practice, obtaining all the necessary data for calculating VO_2_ often poses problems, as while the concentrations of O_2_ leaving and entering the chamber will be known (often given as the concentration of O_2_ in the room air and concentration of O_2_ in the chamber by commercial calorimetry software), only one of the flow rates (either into or out of the chamber) will usually be available. However, it is possible to take advantage of the concentration of nitrogen in the measured gases in order to correct for this. Nitrogen is not produced or consumed by mice, so the flow rate of nitrogen is assumed to be constant. The fractional concentration of nitrogen (FcN_2_) can be calculated by subtracting the fractional concentrations of oxygen, carbon dioxide and water from 1. Of note, if systems scrub (remove) water or CO_2_, only the fractional concentrations of the non-scrubbed gasses must be subtracted from 1 to give the FcN_2_. Given that nitrogen is not produced or consumed, the following equation is valid:

(3) $${\text{FR}}_{\text{in}}{\text{FcN}}_{2\text{in}}={\text{FR}}_{\text{out}}{\text{FcN}}_{2\text{out}}$$

If Equation 3 is rearranged then the flow rate out of the chamber can be calculated from the fractional concentrations of nitrogen and the flow rate into the chamber:

(4) $${\text{FR}}_{\text{out}}={\text{FR}}_{\text{in}}\frac{{\text{FcN}}_{2\text{in}}}{{\text{FcN}}_{2\text{out}}}$$

VO_2_ can now be calculated when only one flow rate is known (in this case FR_in_) by substituting Equation 4 into Equation 2 to yield Equation 5.

(5) $${\text{VO}}_{2}={\text{FR}}_{\text{in}}{\text{FcO}}_{2\text{in}}-{\text{FR}}_{\text{in}}{\text{FcO}}_{2\text{out}}\frac{{\text{FcN}}_{2\text{in}}}{{\text{FcN}}_{2\text{out}}}$$

The VO_2_ will be given in the units that the flow rate is expressed in (i.e., ml/min).

The lag correction itself relies on recalculating the FcO_2out_ in Equation 5. Correcting for lag relies on taking the first derivative of the oxygen concentration between two time points (T_0_ and T_−1_) multiplied by a constant and then adding it to the oxygen concentration at T_0_. This correction is known as the Z correction or instantaneous correction. A simplified form of The Z correction is as follows:

(6) $$\text{K}\left({\text{FcO}}_{{\text{2outT}}_{\text{0}}}-{\text{FCO}}_{{\text{2inT}}_{-\text{1}}}\right)+{\text{FcO}}_{{\text{2outT}}_{\text{0}}}$$

The corrected Fc_out_ shown in Equation 6 for O_2_ can be substituted into the calculation for VO_2_ (Equation 5). In theory the value K should be related to the volume of the chamber, the flow rate and the time between measurements according to the Z equation (Lighton, 2008). However, in practice the constant K should be determined empirically by infusing a square wave signal of gas using an infusion pump, as theoretical chamber volumes often differ substantially from observed chamber volumes. The constant should be adjusted manually until the data produces as close an approximation of a square wave as is possible. Application of such a correction would make the observed signals shown in Figure 2 for the different chamber volumes represent a horizontal line from 0 to 90 min that intercepts the y-axis at 1 W.

Most calorimeters provide gas concentrations in percentages and the following equations are rederivations of the VO_2_ and Z corrections above. Equation 7 provides the VO_2_ at any given time point and Equation 8 provides the differential VO_2_ between a given time point (T_0_) and the previous time point (T_−1_).

(7) $$\text{Flow rate}(\text{ml/min})\frac{\%{\text{N}}_{2\text{out}}}{100}\left(\frac{\%{\text{O}}_{2\text{in}}}{\%{\text{N}}_{2}\text{in}}-\frac{\%{\text{O}}_{2\text{out}}}{\%{\text{N}}_{\text{2out}}}\right)$$

(8) $$\text{C}\frac{\%{\text{N}}_{2{\text{outT}}_{\text{0}}}}{100}\left(\frac{\%{\text{O}}_{2{\text{outT}}_{\text{-1}}}}{\%{\text{N}}_{2{\text{outT}}_{\text{-1}}}}-\frac{\%{\text{O}}_{2{\text{outT}}_{\text{0}}}}{\%{\text{N}}_{2{\text{outT}}_{\text{0}}}}\right)$$

Adding Equation 7 (calculated for T_0_) and Equation 8 together gives the lag-corrected VO_2_ at any given time point in ml/min. In Equation 8, C is a constant that theoretically is the chamber volume divided by the time between T_0_ and T_−1_, with both in the units of the flow rate (e.g., ml for the chamber volume and minutes for time if the flow rate is ml/min). However, as mentioned above, the constant C should be empirically determined. Performing the same calculations for %CO_2_ allows lag corrected values for -VCO_2_ to be calculated. Once lag-corrected VO_2_ and VCO_2_ have been determined, the lag-corrected energy expenditure can be calculated using the Weir equation (Equation 1), and the lag corrected RER can be calculated by dividing VCO_2_ by VO_2_.

## Impact of lag on maximal thermogenic capacity data

**Figure 3:**
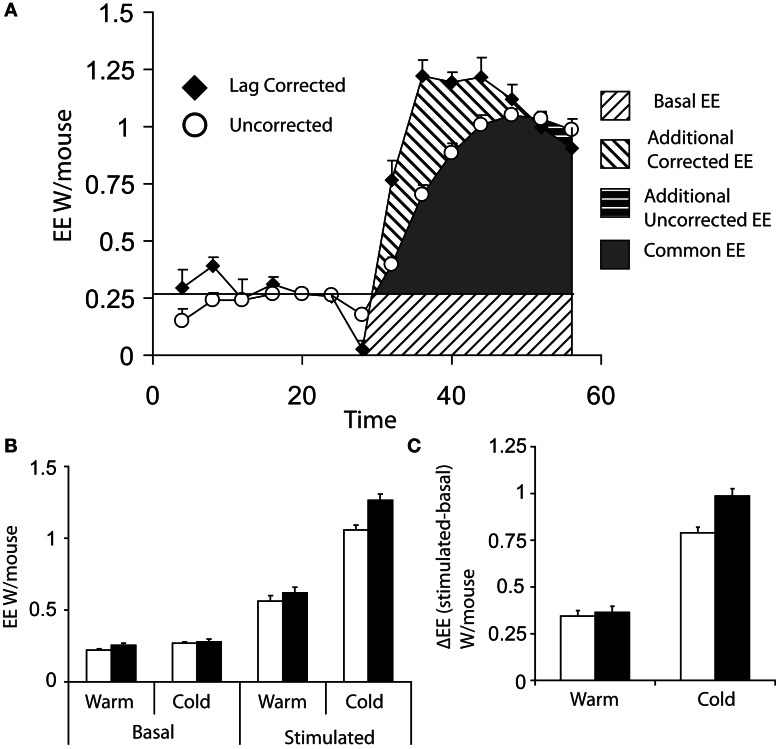
**(A)** Comparison of energy expenditure plots from Figures 1A,B of 8 cold acclimated mice, which have been corrected for lag (black diamonds) or uncorrected (white circles). **(B)** Basal (the average of the last three energy expenditure readings prior to injection) and maximum norepinephrine (NE) stimulated energy expenditure (energy expenditure) (average of the largest three recorded values) based on either lag corrected (black bars) or uncorrected (white bars). **(C)** Δ energy expenditure (maximal stimulated energy expenditure—basal energy expenditure) in response to norepinephrine corrected for lag (black bars) or uncorrected (white bars). All mice; male, C57Bl/6, 4 months of age, chamber volume 2.7 l. Energy expenditure measurements conducted using a custom built calorimetry system, using a paramagnetic oxygen analyser and infrared carbon dioxide detector. Flow rate was 0.4 l/min and measured on incurrent air to the chambers.

Figure 3A shows the difference between lag corrected and lag uncorrected data from Figure 1. As can be seen, the maximal rate of thermogenesis in the uncorrected data is apparently observed at approximately 24 min post injection of norepinephrine. With lag correction, maximal thermogenesis is observed only 12 min post injection. Lag correction is desirable, because chambers of different volumes have different lag periods, thus in different facilities mice may appear to take more or less time to respond to norepinephrine.

Analysis of data from maximal thermogenic capacity is an area that deserves some consideration. In theory the single highest value obtained is the maximal thermogenic capacity; however, it is perhaps prudent to consider the largest three values reached during assessment of maximal thermogenesis in order to have a more stable value. (Lelliott et al., 2006). When analysing maximal thermogenic capacity, lag correction can have a substantial effect. Lag correction of norepinephrine stimulated energy expenditure gives a substantially larger maximal energy expenditure than that observed with uncorrected data (Figure 3B) and the difference in energy expenditure (Δ energy expenditure) between basal and stimulated energy expenditure, particularly in the cold, is increased (Figure 3C).

## The use of conscious vs. unconscious animals for maximal thermogenic capacity measurements

**Figure 4:**
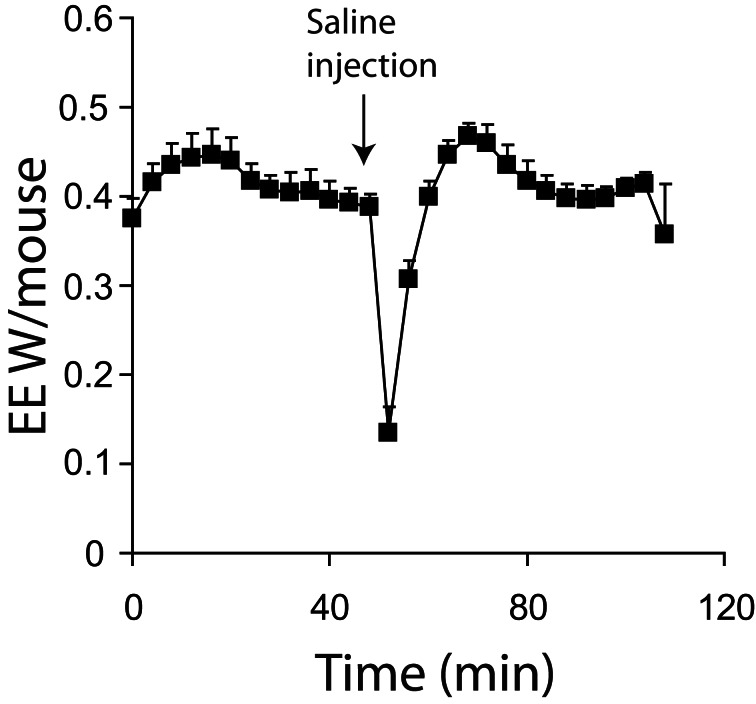
**Energy expenditure of 7 mice acclimated and measured at 21°C.** All mice were conscious during the experiment. Mice were injected with saline where indicted. All mice; male, C57Bl/6, 4 months of age, chamber volume 6.2 l. Energy expenditure measurements conducted using a custom built calorimetry system, using a paramagnetic oxygen analyser and infrared carbon dioxide detector. Flow rate was 0.4 l/min and measured on incurrent air to the chambers.

Maximal thermogenic capacity measurements can be conducted in conscious (Meyer et al., 2010) or unconscious (Golozoubova et al., 2006) mice. There are several practical considerations to either approach. First, injecting conscious animals, even with saline, will result in a relatively large increase in energy expenditure due to handling stress (Figure 4). Additionally, with conscious mice, physical activity may introduce considerable noise into experiments. These problems are diminished in the stimulated state when utilizing potent thermogenic agents such as norepinephrine, as their effect on energy expenditure is much greater than noise from activity or injection stresses. However, obtaining good baseline values for energy expenditure is likely to be problematic in conscious animals. Minimal observed energy expenditure values (for example the lowest 3 energy expenditure readings observed over a 3 h period) are often used as a measure of basal metabolic rate (BMR) (Meyer et al., 2010). The assumption is that the lowest values will represent periods when mice are inactive and thus BMR can be accurately observed. There are two potential problems with using minimal observed energy expenditure values in free living mice. First, if lag is not corrected for, then the minimal observed energy expenditure values are likely to be overestimated as they will include the effects of energy expenditure that have occurred over a 30+ min period dependent on the cage volume. Secondly, if energy expenditure is corrected for lag, there is a potential to underestimate BMR. Use of the Z correction for lag can result in an over estimation of the change in energy expenditure if there is noise from the oxygen analysers (Lighton, 2008), or the constant K (Equation 6) is too large.

Using unconscious animals removes many of the potential problems seen in conscious mice. Baseline measurements become stable more rapidly as endogenous SNS tone is minimized (Figure 1A vs. Figure 4). Noise due to processes such as physical activity is also very low. When using unconscious animals, substantial care must be taken regarding the anesthetic of choice. While most anesthetics lead to a reduction in sympathetic tone in mice (hence the requirement to control body temperature during surgery) it is critical that they do not impair sympathetic signaling. Gaseous anesthetics such as isoflurane are known to disrupt adrenergic signaling at the level of receptors (Ohlson et al., 1994). So far, to our knowledge, the only published anesthetic used in maximal thermogenic capacity measurements is pentobarbital. Pentobarbital has a relatively narrow anesthetic window between full sedation and death. It also seems that cold-acclimated mice are more resistant to the effects of pentobarbital when compared to warm acclimated mice (authors' own observation), making anesthetic dose optimization necessary for any new experimental protocol.

## Sympathetic nervous system tone and the regulation of BAT

The single most important regulator of BAT function is the SNS (Cannon and Nedergaard, 2004). As such, assessing SNS tone in mice is a technique of considerable value.

### Central regulation of sympathetic tone

Considerable work has investigated the pathways that mediate how an organism; (1) detects changes in environmental temperature; (2) relays and integrates information regarding changes in environmental temperature within the central nervous system, and (3) activates thermoregulatory pathways that act to defend core body temperature. A series of excellent reviews have covered central regulation of thermogenesis and fever (Morrison et al., 2008, 2012; Nakamura, 2011).

A series of putative peripheral temperature receptors that belong to the transient receptor potential (TRP) family of cation-selective channels have been identified. TRPA1 (Story et al., 2003) and TRPM8 (Mckemy et al., 2002; Peier et al., 2002) have been suggested to detect noxious and mild cold respectively. Conversely, TRPV1 is suggested to be an extreme heat receptor (Caterina et al., 1997). Finally, TRPV3 (Xu et al., 2002) and TRPV4 (Guler et al., 2002) have been suggested to be able to detect subtle increases in temperature.

Once changes in skin temperature are detected, sensory neurons transmit signals ultimately to the preoptic area of the hypothalamus. The pathway for afferent temperature signals has been shown to be via the dorsal horn (Craig et al., 1994), followed by the lateral parabrachial nucleus (Nakamura and Morrison, 2008). Peripheral temperature signals principally converge on the preoptic area. In addition to signals from peripheral cold receptors, the preoptic area also integrates inflammatory signals, which regulate fever via the production of prostaglandin E2 (Nakamura, 2011) which is sensed by EP3 receptors in the preoptic area (Lazarus et al., 2007). In response to cold, the preoptic area regulates SNS outflow to peripheral vessels, heart and BAT (Morrison et al., 2012). The preoptic area principally regulates sympathetic tone via inhibition of neurons in the dorsomedial hypothalamus (Dimicco and Zaretsky, 2007). Skin cooling attenuates the inhibitory signal from the preoptic area to the dorsomedial hypothalamus (Nakamura and Morrison, 2007), which is then able to activate premotor neurons in the rostral raphe pallidus which lead to BAT activation via activation of sympathetic preganglionic neurons in the spinal intermediolateral cell column (Morrison et al., 2012).

Ultimately, the critical effector arm of the nervous system for control of BAT is the SNS. Therefore, determining the outflow of the SNS to BAT is of considerable value for identifying the potential mechanistic basis of differences in BAT capacity or function.

## Measurement of sympathetic tone

Three major methods for directly measuring sympathetic tone exist, while there are also several indirect molecular-markers of sympathetic tone that can be used to assess tissue-specific sympathetic activity. In this section we will discuss practical considerations regarding these methods.

## Direct methods for assessment of sympathetic nervous system tone

### Direct nerve recording

Direct nerve recording is based on surgically attaching electrodes to nerves subtending BAT (or any other tissue of interest) and recording nerve firing over time (Morrison, 1999; Rahmouni et al., 2005). Once electrodes are attached, recordings can be carried out over period of up to 3 h, during which time stimuli designed to modulate sympathetic nerve signaling can be applied. One such stimulus is to cool the anaesthetized mouse in order to obtain a “cold exposure” response (Morrison, 1999; Whittle et al., 2012). Equally, responses to administration of drugs, either peripherally or directly to specific brain regions via intracranial injection (IC), can be assessed in terms of alterations in SNS tone. By using a combination of the pattern of SNS firing and site-specific IC injections of inhibitors and activators of SNS tone it is also possible to determine which brain regions are responsible for regulating SNS tone to specific tissues and organs (Morrison, 1999). One of the key aspects of such studies is that both sympathetic firing and physiological responses can be simultaneously monitored, with changes in blood pressure heart rate and energy expenditure monitored at the same time as SNS outflow over a time scale of seconds. Direct nerve recordings have been of particular value for investigating the brain regions involved in central control of thermogenesis. For example, as mentioned above, the dorsomedial hypothalamus receives inhibitory inputs from the preoptic area which are alleviated in response to cold. Activation of the dorsomedial hypothalamus promotes activation of BAT via glutamatergic signaling to the rostral raphe pallidus, which in turn activates downstream signals that ultimately converge on BAT. The presence of a tonic γ-aminobutyric acid A Receptor (GABA_A_) mediated inhibition of neurons in the dorsomedial hypothalamus and its role in controlling BAT SNS outflow was demonstrated by injection of the GABA_A_ agonist muscimol into the dorsomedial hypothalamus, which prevents SNS outflow to BAT in response to skin cooling (Nakamura and Morrison, 2007). Conversely, injecting the GABA_A_ receptor antagonist bicuculline into the same nucleus causes increased SNS outflow to BAT (Zaretskaia et al., 2002).

Direct nerve recordings have some very powerful advantages over other methods of assessing SNS tone. Direct nerve recordings have a far higher temporal resolution than other techniques for assessing SNS tone, which has allowed the central pathways regulating BAT function to be mapped out. Additionally, they also provide information about the nature of nerve firing events (frequency and amplitude). As different tissues within organs can possess different SNS activation patterns (Morrison, 2001) direct recordings of single nerve fibers can allow high resolution analysis of SNS activity to a tissue and potentially allow assignment of SNS outflow to specific functions within the tissue.

Direct nerve recordings are limited by several practical considerations. First, recordings can only be performed on anaesthetized animals, limiting the range of physiological stimuli that can be investigated. Second, an expensive electrical recording set up is required. Comprehensive electromagnetic isolation is required in order to allow detection of the extremely low voltages generated by nerves, requiring a dedicated space within an animal facility. Equally a very high degree of technical skill is required to enable appropriate electrode placement. Overall, direct nerve recordings currently remain the provision of dedicated laboratories.

### Norepinephrine turnover using radioactive tracers

Norepinephrine turnover relies on the fact that sympathetic nerve endings reuptake norepinephrine, allowing exogenously administered radioactively-labeled norepinephrine to be accumulated in tissues. The first step in the norepinephrine turnover method for assessing SNS tone is to administer radio-labeled norepinephrine at tracer levels intravenously (IV) to a mouse prior to study. Norepinephrine is then taken up by nerve endings along with endogenous norepinephrine as part of the norepinephrine reuptake system. Following a wash-out period (where excess norepinephrine is lost in urine), groups of animals can be exposed to a given stimuli (e.g., cold) and the rate of loss of labeled norepinephrine from the tissue at different time points can be assessed. Norepinephrine is lost from the tissue when nerves fire, as reuptake of norepinephrine is not 100% efficient. Lost norepinephrine is replaced by newly synthesized and therefore unlabelled norepinephrine. Thus, as sympathetic nerves fire, the tissue-levels of norepinephrine decrease over time and the rate of norepinephrine depletion is assumed to be proportional to the rate of sympathetic nerve firing. This technique assumes that the percentage rate of norepinephrine reuptake is constant across the animals studied, although baseline differences in reuptake can in part be accounted for by the starting levels of labeled norepinephrine within a tissue, as the amount of label initially entering the tissue will be proportional to reuptake. The total amount of norepinephrine in the tissue must be assessed in order to provide mass rates of norepinephrine turnover (i.e., ng/organ/hour) as opposed to purely providing rates of turnover as percentages per hour. Importantly, calculations for the turnover method rely on there being a steady state level of norepinephrine within the tissue. To confirm norepinephrine levels are stable, norepinephrine concentrations in the tissues must be measured at each time point. Measurement of catecholamines has been reviewed extensively elsewhere (Peaston and Weinkove, 2004) and is non-trivial, particularly when using tissue extracts. Isolation of catecholamines is usually performed by acid washed alumina extraction and the concentration is determined either by mass spectrometry (GC-MS) or high performance liquid chromatography (HPLC) coupled to electrochemical detection (Maycock and Frayn, 1987). Although ELISA techniques exist they tend to lack sensitivity compared to GC-MS and HPLC methods.

Measuring sympathetic tone using radioactive tracers has one major advantage over direct sympathetic nerve recordings—it can be performed in conscious animals over periods of time up to at least 24 h. This means that the potential for suppression of endogenous SNS tone by anesthetic is eliminated when compared to the direct nerve recording method. Equally, the effect of physiological challenges such as high-fat feeding can be assessed while the stimulus is actually occurring, as opposed to only studying adaptive responses when using direct nerve recordings.

Conversely, measuring sympathetic tone by norepinephrine turnover has several major disadvantages over direct nerve recordings. Firstly, it is animal intensive; each time point requires a group of animals. Even using a small group (e.g., *n* = 4) for each time point will require at least 12 animals per intervention (e.g., 12 WT and 12 KO) because three time points are necessary. The requirement for three or more time points comes from the fact that norepinephrine turnover should follow a semi-exponential decay profile and 3 time points is the minimum possible to assess if the data fits this profile. Secondly, the capacity to house radioactive, conscious animals within an animal facility is required. The quantity of radioactivity is also substantial—18 to 37 MBQ per kg being a typical level of tracer. Third, the tracer method relies on a steady state level of norepinephrine. For example, acute cold exposure for 24 h will reduce endogenous BAT norepinephrine content by half—invalidating the use of the tracer method (Young et al., 1982). To confirm that norepinephrine levels are at steady state during an experiment, radioactive samples have to be analysed by HPLC or GC-MS requiring an expensive and potentially dedicated piece of equipment.

### Sympathetic tone assessment by tyrosine hydroxylase inhibition

The final method routinely used for measuring SNS activity is the tyrosine hydroxylase inhibition method. Tyrosine hydroxylase inhibitors such as alpha methyl-P-Tyrosine (AMPT) methyl-ester hydrochloride are used to block synthesis of new catecholamines including norepinephrine. Again this technique relies on the fact that norepinephrine reuptake by sympathetic nerve endings is less than 100% efficient. Blocking synthesis of new catecholamines leads to depletion of norepinephrine in the nerve endings within BAT and the rate of disappearance of norepinephrine from the tissue is assumed to be proportional to the rate of sympathetic nerve firing. The fractional turn over rate (based on the slope of the disappearance) is multiplied by the initial norepinephrine level of the baseline group in order to obtain the norepinephrine turn over rate (Young et al., 1982).

The use of AMPT inhibition has almost the same advantages and disadvantages as the tracer technique when compared to the direct nerve recording method. However, unlike the tracer method described above, the inhibitor method has the advantage of being compatible with experiments where there is a non-steady state level of norepinephrine (Young et al., 1982). The major disadvantage of the inhibitor method when compared to the tracer method is that it can only be used for relatively short periods of time (usually up to 6 h dependent on physiological conditions) as over prolonged periods of time tissue norepinephrine stores will be depleted. Furthermore, AMPT reduces tissue catecholamine levels in all tissues, potentially affecting the phenotype under study. Overall, the radioactive tracer method is preferable to the tyrosine hydroxylase inhibition method except in cases where endogenous norepinephrine levels in the tissue under study are not stable.

### Non-specific methods of assessing sympathetic activity

There are several simple methods that can indicate potential alterations in SNS outflow to BAT. Firstly, measurement of molecular markers of SNS activity in BAT can be used to assess adrenergic signaling. Acute changes in the phosphorylation of proteins such as cAMP response element binding protein (CREB), HSL and p38 mitogen activated protein kinase (p38 MAPK) are all good markers of increased β-adrenergic signaling. An important caveat is that many of these proteins will have their phosphorylation status actively down-regulated after chronic exposure, making the time point studied critical. Measuring the phosphorylation status of proteins in response to adrenergic stimulation has the advantage that it is practically straightforward, particularly given the commercial availability of good antibodies. However, as mentioned above, assessing phosphorylation status of proteins tends to be applicable only to acute interventions. Longer-term markers of alterations in SNS tone include mRNA markers of thermogenic genes such as UCP1, Elongation of very long fatty acids 3 (Elovl3), Deiodinase 2 (Dio2), Peroxisome proliferator-activated receptor gamma coactivator 1α (PGC1α), Bone morphogenetic protein 8b (BMP8b) and lipocalin prostaglandin D synthase (L-PGDS). Importantly, mRNA or protein markers can only provide information about potential changes in the capacity of BAT to generate heat; they do not provide information about how much heat is actually being produced. A second important caveat is that, changes in mRNA expression levels for genes such as UCP1 or PGC1α do not necessarily correlate directly with physiological changes in BAT thermogenic capacity. (Nedergaard and Cannon, 2013). Finally, many genes induced by SNS activation can also be regulated by non-SNS signals such as thiazolidinediones or changes in thyroid hormone levels, making their analyses alone only suggestive of elevated SNS tone.

### Beta adrenergic antagonists

Beta adrenergic antagonists can be applied to mice to assess if physiological variables such as energy expenditure or molecular markers (see above) of BAT activation are sympathetically mediated. While simple and rapid, beta adrenergic agonists are non-specific and will affect multiple tissues. Furthermore, they will not necessarily distinguish alterations in SNS tone from post-receptor changes in sensitivity to sympathetic tone. Nevertheless, they are simple, cheap and quick and will implicate the SNS at some level in modulating a given phenotype.

## Alternative specialized measures of BAT activation

### Thermography and temperature probes

BAT produces heat; therefore it is attractive to think that directly measuring heat production in BAT would be a good method for assessing thermogenesis. On a qualitative level this is probably true, as thermogenic agents such as norepinephrine (Hardman and Hull, 1970; Christoffolete et al., 2004), the TRPA1 agonist allyl-isothiocyanate (AITC) (Masamoto et al., 2009), prostaglandin E2 (Nakamura et al., 2002) and bone morphogenetic protein 8b (Whittle et al., 2012) are able to increase BAT temperature as measured by probes implanted into BAT (Hardman and Hull, 1970; Christoffolete et al., 2004; Masamoto et al., 2009) or by thermography of the skin overlaying the interscapular BAT depot (Whittle et al., 2012). Critically, however, neither method is likely to give a good quantitative measure of thermogenesis.

Infusion of norepinephrine has been reported to increase interscapular BAT temperature by 2°C (Hardman and Hull, 1970) or 6°C (Christoffolete et al., 2004). Norepinephrine infusion can increase metabolic rate by up to 300%, dependent on the prior temperature acclimation of the animal. However, not all changes in BAT temperature are linked to changes in metabolic rate. Intragastric administration of AITC causes an increase in BAT temperature of 1°C (Masamoto et al., 2009). However, subsequent measurements of energy expenditure in response to AITC demonstrated no alterations in total energy expenditure (Mori et al., 2011). AITC also causes vasoconstriction (Masamoto et al., 2009), suggesting that the alteration in BAT temperature could be as a result of increased thermal insulation rather than BAT activity. Furthermore, while a 6°C increase in BAT temperature was observed with norepinephrine infusion, this was assessed over only a 60 min period (Christoffolete et al., 2004). It is unlikely that BAT can maintain such a large increase in tissue temperature as this would eventually kill the tissue. Instead, it is probable that alterations in BAT blood flow, which increases more than 4 fold during norepinephrine infusion in rabbits (Hardman and Hull, 1970) and 25 fold in rats (Foster and Frydman, 1978), would ultimately act to reduce BAT temperature. Several practical considerations regarding the use of temperature probes must be considered. Firstly, an increase in BAT temperature should both precede an increase in core body temperature and should also exceed the temperature of the core, in other words there should be a temperature gradient from warmer BAT to a cooler core. An example of a change in BAT temperature preceding a change in core body temperature can be seen in response to chemical stimulation of the dorsomedial hypothalamus (Zaretskaia et al., 2002). If BAT temperature alone is measured, it is important to consider that a change in BAT temperature may occur due to peripheral vasoconstriction or non BAT-mediated increases in core body temperature.

Overall, while an increase in BAT temperature relative to core body temperature indicates that BAT has been activated; it does not provide reliable quantitative information about the degree of activation.

### Fluorodeoxyglucose (^18^F) positron emission tomography (FDG-PET)

The use of FDG-PET to assess BAT has come into focus since the rediscovery of BAT in adult humans (Hany et al., 2002; Nedergaard et al., 2007; Cypess et al., 2009; Saito et al., 2009; van Marken Lichtenbelt et al., 2009; Virtanen et al., 2009). FDG-PET allows the assessment of glucose uptake into tissues *in vivo*. Deoxyglucose is an analogue of glucose that is taken up by glucose transporters but cannot be metabolized. Once taken up, deoxyglucose is phosphorylated and becomes trapped in the tissue, making its levels within a tissue proportionate to glucose transport. FDG-PET has been used in a number of rodent studies to investigate the effects of both pharmacological activators of BAT including CL316243 (Mirbolooki et al., 2011), nicotine and ephedrine (Baba et al., 2007) as well as acute cold exposure for 2–4 h (Baba et al., 2010; Mirbolooki et al., 2011). While FDG-PET does show some dose dependence in response to CL316243 (Mirbolooki et al., 2011), the relationship between FDG-PET uptake and energy expenditure has not been assessed in rodents. The effect of temperature on FDG uptake into BAT has only be assessed for rats in response to acute cold exposure, with 2 h at 8°C causing barely any increase in glucose uptake (Mirbolooki et al., 2011) whereas 4 h at 4°C causes a doubling of glucose uptake (Baba et al., 2010). Importantly, in both the studies of Mirbolooki et al. and Baba et al. BAT thermogenesis would be expected to occur, highlighting the fact that FDG-PET only allows assessment of glucose uptake which may not mirror thermogenic activity.

While FDG-PET is a potentially valuable technique allowing simultaneous assessment of separate BAT depots within the same organism, FDG-PET has several major limitations. First, due to size limitations FDG-PET studies have focused almost exclusively on rats and larger animals. More importantly, at present no studies have demonstrated a correlation between energy expenditure and FDG uptake into BAT in mice. Furthermore, FDG-PET is very expensive, requiring both access to an appropriate scanner, the ability to synthesise ^18^F-FDG and the capacity to very rapidly transport ^18^F-FDG to the site where the animal is under study, given the 109.8 min half life of ^18^F. Given these limitations, at present, FDG-PET can only be considered as a qualitative and somewhat expensive method to detect BAT activation in rats and larger animals.

## General techniques for assessing BAT function

The techniques for assessing BAT function described so far are not universally available and tend to be restricted to specialized units. However, a number of simple assessments described below can provide helpful indicators of potential alterations in BAT function and thermogenic capacity.

### Bat weight

**Figure 5:**
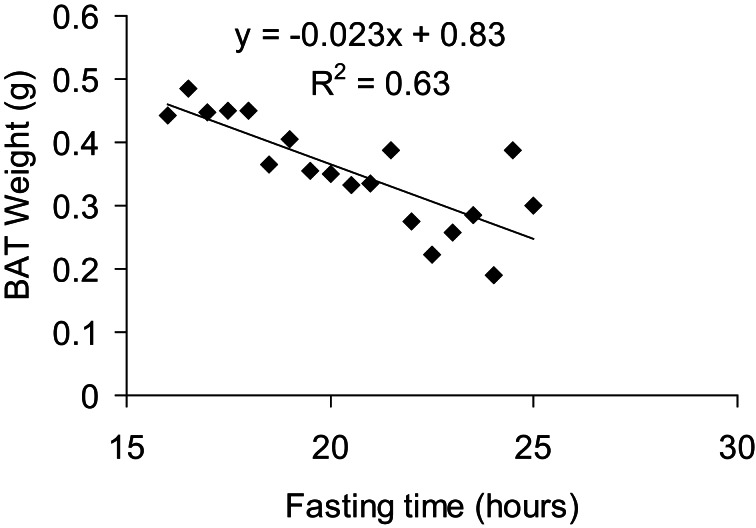
**Correlation between BAT weight and fasting time.** All mice were fasted overnight for 15 h and then dissected over a period of 8 h, giving a total fasting time ranging from 16 to 24 h for the animals studied. Mice were 7 months old, C57Bl/6 males fed a 60% HFD and acclimated to 5°C for at least 3 weeks prior to culling.

BAT weight is a complex variable as it can be affected by both long term factors such as alterations in the number of brown adipocytes, but also by acute factors such as nutritional status. As such interpreting BAT weight in the absence of other information (histology, gene expression, and metabolic data) is virtually impossible and its value as anything other than a qualitative variable (i.e., something happened in BAT) is of question. As mentioned below in the histological analysis section, the lipid content of BAT is highly plastic. Acute cold exposure will entirely delipidate BAT, reducing the tissue's weight while its activity is very high (Christoffolete et al., 2004). Conversely, fasting will also reduce BAT weight (Figure 5) while its activity is actually diminished. Nevertheless, alterations in BAT weight are simple to assess and should be considered as a marker of potential alterations in BAT, worthy of further investigation.

### Pair feeding

Pair feeding is a powerful technique for detecting differences in metabolic rate, particularly where groups of animals have differences in food intake and differences in body weight. Pair feeding is usually conducted by feeding the hyperphagic group the same amount of food as the hypophagic group ate the previous day. If pair feeding does not fully correct a difference in body weight between the groups it is indicative of an alteration in metabolic rate.

When conducting pair feeding it is important to be able to accurately measure food intake. While this sounds simple mice often grind their food, reducing it to fine dust which cannot easily be seen amongst rodent bedding. To accurately assess food intake, replacing bedding with blotting paper (to absorb urine but allow collection of powdered food) and the use of food cups is desirable. Tung et al. describe such a method for analysis of food preference, however, it can also be used for accurate assessment of food intake when only one choice of diet is presented (Tung et al., 2007). However, even a difference in body weight gain between groups of animals that have been pair fed only implies there is altered energy expenditure; whether it is BAT mediated must be confirmed by additional experiments.

### Response to fasting

Weight loss in response to fasting is another potential method for assessing changes in metabolic rate. A greater weight loss in response to an over-night fast is indicative of a hypermetabolic phenotype. In some respects, this is a subset of the pair feeding experiments described above; in this case both groups of animals receive no food instead of the same amount. Again, it is important to note that greater weight loss in response to fasting does not by itself indicate an alteration in BAT function as other organs could be responsible for the observed hypermetabolism.

### Changes in core body temperature

The immediate effect of transferring a warm acclimated animal to a cold environment is dependent on (1) the magnitude of the temperature change (2) the species (3) the time of day the transfer occurs [due to the known circadian effects on BAT (Redlin et al., 1992)] and (4) the nutritional status of the animal (Meyer et al., 2010). In general, the effect of transferring mice and rats at any temperature to a substantially colder environment (drop in temperature of 12°C or more) over the first hour is either no reduction in core body temperature or a slight increase of between 0.25 and 1°C (Lomax et al., 1964; Golozoubova et al., 2001; Bratincsak and Palkovits, 2005; Meyer et al., 2010). The slight increase in core body temperature occurring within the first hour is assumed to be related to the combination of increased thermogenesis and peripheral vasoconstriction, which reduces peripheral heat loss.

Over the subsequent 3–4 h of acute cold exposure the response of an animal in terms of core body temperature is heavily dependent on its thermogenic capacity (both shivering and NST). Wild-type mice that have been acclimated to 18°C prior to cold exposure can maintain their core body temperature to within 2°C when exposed to an ambient temperature of 5°C (Golozoubova et al., 2001). When moved to 5°C, wild-type mice that have previously been acclimated to 30°C show a large drop in core body temperature to around 26°C by 3 h (Golozoubova et al., 2001). Mice lacking UCP1 when acclimated to 24°C and then transferred to 5°C rapidly reduce core body temperature, reaching 27°C within 3 h, whereas wild-type mice previously acclimated to 24°C are able to maintain core body temperature at 37°C (Golozoubova et al., 2001). Overall, these results suggest that mice with low thermogenic capacities (either wild-type mice acclimated to 30°C or UCP1 KO mice) do not defend their core body temperature during acute cold challenges as well as mice with greater thermogenic capacities (wild-type mice acclimated to 24°C). However, while a failure to defend core body temperature appropriately can indicate a lack of thermogenic capacity, it is important to note that substantial drops in core body temperature can occur in a regulated manner via the process of torpor. Torpor is an energy preserving state in which mice reduce their core body temperature to as low as 19–20°C and their metabolic rate by 50%. Importantly, mice can spontaneously recover from bouts of torpor lasting several hours (Meyer et al., 2010).

A reduction in core body temperature in response to acute cold exposure is not necessarily a marker of impaired BAT thermogenesis. An inability to undergo shivering thermogenesis can also impact on core body temperature in response to acute cold exposure. The gene sarcolipin has recently been shown to be necessary for appropriate shivering in muscle. Mice lacking the gene sarcolipin, accompanied with surgical removal of interscapular BAT, cannot tolerate cold exposure (Bal et al., 2012) and have a more rapid and sustained loss in core body temperature compared to wild-type mice which have had their interscapular BAT surgically removed.

In general, unless investigation of torpor is a central aim of the study, a core body temperature drop of 10°C is a sensible endpoint for safe termination of an acute cold exposure experiment. From a practical point of view, if an intervention group exhibits a substantially greater loss in core body temperature over a period of 4 h it is sufficient to indicate a potential issue with thermogenesis; however, the involvement in BAT in this process must be determined.

### Bat lipid content

Acute cold exposure leads to a rapid delipidation of BAT as mobilization and oxidation of endogenous lipid stores outstrips the capacity of brown adipocytes to take up or synthesise lipids (Christoffolete et al., 2004). The first physiological adaptation to cold exposure in BAT is an increase in lipid uptake. On a transcriptional level, expression of the gene lipoprotein lipase (LPL), which is essential for lipid uptake into BAT, is induced as early as 1 h after cold exposure (Mitchell et al., 1992).

Subsequently, *de novo* lipogenesis rates increase, with a significantly increased *de novo* lipogenic capacity detectable at 24 h (Christoffolete et al., 2004). On a gene expression level, at 24 h post-cold exposure there is an increase in glycolytic genes (Yu et al., 2002) and the enzyme acetyl-CoA carboxylase (ACC), which produces the fatty acid synthesis intermediate malonyl-CoA (Yu et al., 2002; Christoffolete et al., 2004). Interestingly, fatty acid synthase (FAS) or SCD1 are not induced as early as ACC, suggesting that levels of ACC in BAT may be the factor limiting *de novo* lipogenic rates.

By 7 days post cold exposure BAT has become largely restocked with lipid, with lipid droplets returning to almost the same size as warm acclimated mice (Christoffolete et al., 2004). By 3 weeks post cold exposure both SCD1 and FAS are induced over control levels. These gene expression changes suggest that BAT increases the rate of lipid restocking of adipose tissue stores and in the longer term allows a maintained high rate of lipid uptake and *de novo* synthesis of lipids.

Brown fat lipid content can be modulated by pharmacological and genetic interventions that impact on BAT in a variety of ways. Treatment with thermogenic compounds such as CL316243 can result in substantial reductions in BAT lipid content within just 2 h (Mirbolooki et al., 2011). Conversely, the UCP1 KO mouse exhibits greater BAT lipid content than wild-type mice at room temperature (21–24°C), assumed to be because of reduced oxidation rates (Enerback et al., 1997).

However, not all changes in BAT lipid content are related to alterations in the metabolic rate of BAT. Lipocalin-Prostaglandin D Synthase KO mice exhibit greater lipid content in BAT under cold exposed (4°C) conditions and express markers of elevated *de novo* lipogenesis in BAT when compared to wild-type controls, despite similar levels of maximal thermogenic capacity (Virtue et al., 2012b). Finally, the elongation of very long chain fatty acids protein 3 (Elovl3) KO mouse exhibits similar lipid content in BAT after acute (3 days) or chronic (3 weeks) exposure to 4°C but has much lower lipid content after housing for 3 weeks at 30°C (Westerberg et al., 2006). The altered lipid level in the BAT of Elovl3 KO mice is complex to interpret. Elovl3 KO mice exhibit altered lipid metabolism and reduced body weight at room temperature (Zadravec et al., 2010) as well as a striking impairment in fur development and skin function (Westerberg et al., 2004). Both these factors could impact on BAT lipid content at 30°C. Firstly, 30°C may not represent thermoneutrality in these mice due to lower insulation. Alternatively, their reduction in peripheral fat mass may affect BAT lipid storage when BAT is inactive at higher temperatures, whereas when BAT must be active (at 4°C) BAT lipid metabolism is spared compared to other organs.

Practically, BAT lipid content can be assessed either by histological morphometry or biochemically. Haematoxylin and eosin staining will stain all non-lipid areas of BAT. Lipid droplets will appear white. Analysing the total white area of a section of BAT by phase analysis will give % lipid content per section. Alternatively, biochemical techniques such as Folch extraction can be used to assess total BAT lipid content (Mclaughlin et al., 2010). The Folch technique relies on accurately weighing BAT and then extracting all the lipid using solvents (chloroform:methanol) before evaporating the solvents and weighing the lipid.

Overall, there are two major considerations for the analysis of BAT lipid content. First, given the dynamic alterations in BAT lipid accumulation in response to cold exposure, it is important to consider the environmental temperature mice were previously exposed to and for how long they were exposed to it. Second, given that multiple different biological processes can affect lipid levels within BAT, assessment of BAT lipid content cannot be considered indicative of alterations in BAT function when taken in isolation.

### Gene expression markers

Multiple mRNA markers for activation of BAT and for the browning of WAT exist. The most well-established BAT marker by far is UCP1. It is again important to state that changes in mRNA or even levels of protein markers in BAT will only provide information about the potential thermogenic capacity of BAT, not its activity.

#### Uncoupling protein 1 (UCP1)

UCP1 is essential for thermogenesis by BAT and its expression is strongly regulated by cold exposure, with UCP1 mRNA expression increasing 10 fold between BAT from animals acclimated to 30°C vs. those acclimated to 4°C for 6 days (Madsen et al., 2010). UCP1 expression is induced in BAT within 3 h of cold exposure (Christoffolete et al., 2004). Acute induction of UCP1 expression seems to be predominantly regulated by adrenergic agonists, with the β3-adrenergic receptor-agonist CL316243 able to increase UCP1 expression 6 fold within 4 h (Cao et al., 2001). Importantly, UCP1 mRNA expression is usually expressed as a concentration per μg of RNA. Assessed in this manner UCP1 mRNA peaks after 4 h in the cold and subsequently diminishes after 1–2 days (Nedergaard and Cannon, 2013). As such changes in UCP1 mRNA alone should be treated with caution. A more physiological measure of BAT thermogenic capacity is total UCP1 protein in a specific BAT depot (Nedergaard and Cannon, 2013).

#### Elongation of very long chain fatty acids 3 (Elovl3)

Elovl3, also known as Cold Inducible Gene 30 (Cig30) is the most inducible gene with respect to temperature changes in BAT. Cig30 is induced by as much as 200 fold in the BAT of mice after a 3 days exposure to 4°C, following previous acclimation to 30°C (Tvrdik et al., 1997). The function of Elovl3 with respect to BAT function remains unclear due to skin barrier dysfunction in this model (Westerberg et al., 2004).

#### Peroxisome proliferator-activated receptor gamma coactivator 1α(PGC1α) and PGC1β

PGC1β is induced around 3 fold in BAT when comparing mice acclimated to 4°C with mice acclimated to 30°C, whereas PGC1α is induced 5 fold (Lelliott et al., 2006). PGC1α is a key transcriptional co-activator in BAT that regulates mitochondrial biogenesis and brown adipocyte differentiation. PGC1α has been demonstrated to be necessary for the expression of the BAT thermogenic and differentiation program (Lin et al., 2004). PGC1β is also necessary for cold adaptation of BAT (Lelliott et al., 2006). Ablation of PGC1β impairs maximal thermogenic capacity and results in altered mitochondrial morphology (Lelliott et al., 2006). Loss of both PGC1β and PGC1α is lethal, resulting in mitochondria which lack most of their cristae and therefore have greatly diminished capacity for oxidative phosphorylation (Lai et al., 2008).

#### Deiodinase 2

Deiodinase 2 is an enzyme that converts the thyroid hormone T4 into the active form T3. Thyroid hormone is necessary for full BAT activation and development. Deiodinase 2 is up regulated in states of high BAT activation and is induced by 6 days acclimation to cold exposure by approximately 10 fold (Madsen et al., 2010). Loss of Deiodinase 2 results in impaired capacity for BAT thermogenesis (de Jesus et al., 2001).

## Subcutaneous white adipose tissue and brite cells

A large amount of recent interest has focused on brite cells. These are non-canonical brown adipocytes located within predominantly “white” adipose tissue depots. They are developmentally distinct from brown adipocytes found in canonical “brown” depots such as the interscapular depot. Several agents and physiological conditions have been found to promote the development of brite cells including PPARγ activation (Petrovic et al., 2010), exercise (Cao et al., 2011; Bostrom et al., 2012), bone morphogenetic protein 7 (Schulz et al., 2011) and cold exposure. Conversely loss of PPARγ2 greatly reduces brite cell markers in subcutaneous WAT (Virtue et al., 2012c).

While brite cells have been shown *in vitro* to retain much of the functional capacity of a canonical brown adipocyte (Petrovic et al., 2010), a critical and unresolved question is whether they have any impact on whole-organism energy expenditure. As a tissue, even after cold exposure, the inguinal subcutaneous WAT depot expresses only 10% of the UCP1 of the canonical interscapular BAT depot. In mesenteric or epididymal WAT depots the levels of UCP1 are even lower (Nedergaard and Cannon, 2013).

Even extrapolating function from UCP1 mRNA levels, which represents the best-case scenario, it seems improbable that brite cells could contribute to more than a small proportion of total BAT-mediated thermogenesis. However, mRNA or even UCP1 protein levels are only a surrogate for BAT activity. Whether brite cells have sufficient blood supply and innervation to actually produce heat to a meaningful extent *in vivo* remains undetermined. Until such time as the ability of brite cells to actually contribute to whole-body energy expenditure has been demonstrated, considerable caution must be taken before assigning alterations in brite cell number to alterations in energy expenditure.

One important point regarding brite cells is that changes in BAT markers in subcutaneous WAT have a greater dynamic range than those in canonical BAT (Nedergaard and Cannon, 2013). Analysing markers of BAT in subcutaneous WAT may provide a more sensitive readout of changes in sympathetic tone and/or BAT differentiation than analysis of canonical BAT. Therefore, changes in brite cell number/gene expression should not be ignored as they may be markers of proportionately smaller, but biologically more significant alterations in canonical BAT.

## Discussion

This review has attempted to cover practical and theoretical considerations for the study of BAT. Regardless of which field of metabolic research is under investigation, an understanding of the potential for alterations in BAT function to impact on a given phenotype is essential to allow proper interpretation of results in rodent experiments. The capacity for BAT to clear both lipids and glucose from the circulation and to protect animals from high-fat diet induced obesity means that BAT activity affects all aspects of cardiovascular, diabetes, and obesity research. Regardless of whether BAT activation is ever a successful treatment for human metabolic disease, unless BAT and its metabolic consequences are fully understood then it will be almost impossible to translate data from mouse models into humans.

### Conflict of interest statement

The authors declare that the research was conducted in the absence of any commercial or financial relationships that could be construed as a potential conflict of interest.
